# Serious Game for Fine Motor Control Rehabilitation for Children With Epileptic Encephalopathy: Development and Usability Study

**DOI:** 10.2196/50492

**Published:** 2023-10-03

**Authors:** Elizabeth Vidal, Eveling Castro-Gutierrez, Robert Arisaca, Alfredo Paz-Valderrama, Sergio Albiol-Pérez

**Affiliations:** 1 Universidad Nacional de San Agustín de Arequipa Arequipa Peru; 2 Aragón Health Research Institute (IIS Aragón) Universidad de Zaragoza Teruel Spain

**Keywords:** serious game, virtual motor rehabilitation, ecologic virtual system, fine motor rehabilitation, virtual reality, rare diseases, children with epileptic encephalopathy

## Abstract

**Background:**

Epileptic encephalopathy (EE) is defined as the presence of frequent epileptiform activity that adversely impacts development, typically causing the slowing or regression of developmental skills, and is usually associated with frequent seizures. One of the main disturbances in EE is in the coordination of the upper extremities and hands. Traditional rehabilitation for this type of pathology focuses on the alleviation of gross or fine motor disability. In the last few years, the use of low-cost devices together with customized serious games has shown improvements in motor disorders and enrichments in activities of daily living.

**Objective:**

This study aims to explore the feasibility of a new serious game for improving fine motor control in children with EE.

**Methods:**

The participants were 4 children with EE (male: n=2, 50%; female: n=2, 50%) who were classified as belonging to level 1 in the Gross Motor Classification System. The children were tested over 10 sessions during the intervention period (before and after treatment). The clinical tests performed were the Bruininks-Oseretsky Test of Motor Proficiency, 2nd edition and Pittsburgh Rehabilitation Participation Scale. The subscales of the Bruininks-Oseretsky Test of Motor Proficiency, 2nd edition were fine motor precision, fine motor integration, manual dexterity, and upper-limb coordination. At the end of the first session, we used the User Satisfaction Evaluation Questionnaire to analyze user satisfaction.

**Results:**

The significance outcomes for a Student *t* test (1-tailed) were as follows: *P*=.009 for fine motor precision, *P*=.002 for fine motor integration, *P*=.56 for manual dexterity, and *P*=.99 for upper-limb coordination. The participation rate as measured using the Pittsburgh Rehabilitation Participation Scale was between good and very good, which means that, based on the therapist’s evaluation, interest, independence, and motivation were achieved by each participant. The mean User Satisfaction Evaluation Questionnaire score was close to 30, which is the maximum value.

**Conclusions:**

The results support the use of the proposed serious game as a complement in therapeutic sessions during the rehabilitation processes for children with EE. Significant improvements in fine motor control and activities of daily living revealed that the proposed serious game is beneficial for fine motor disorders of this pathology.

## Introduction

### Background

Fine motor ability refers to the ability of an individual to make precise, voluntary, and coordinated movements with their hands [1]. Fine motor control involves fine motor precision (FMP) and fine motor integration (FMI) [2]. The literature shows that there are fine motor disabilities in pathologies such as Parkinson disease, acquired brain injury, cerebral palsy, and epileptic encephalopathy (EE) [3-6].

Rare diseases are a type of pathology with a low incidence and prevalence worldwide [7]. These diseases with genetic components are characterized by alterations at the motor, cognitive, emotional, and social levels [8]. EE is one of these diseases. This pathology is defined by the presence of frequent epileptiform activity that adversely impacts developmental skills [6,9].

The abundant epileptiform abnormalities and high number of epileptic seizures in EE contribute to cognitive regression [10]. Usually, EEs manifest during early infancy or childhood [6]. Most patients with EEs are recognized to have etiologies due to genetic variants [11,12]. Other etiologies associated with EE include structural, metabolic, and immune disorders [6,13]. Disease expression for different genetic mutations may vary in the type and severity of subsequent epilepsies [14,15].

Rehabilitation methods for children with EE are focused on medications. In general, interventions to control seizures do not change the baseline level of functioning; however, reducing the seizure burden leads to an improvement in quality of life [9]. Other treatment modalities include sodium channel blockers and a ketogenic diet [16]. There is no previous experience with a serious game focused on fine motor rehabilitation.

Childhood EE presents psychomotor alterations with dysfunctions in postural control, tremors, involuntary muscle coordination, motor control problems, and cognitive alterations [17].

Rehabilitation is used to address the problems associated with fine motor control. Common rehabilitation exercises include a conventional physiotherapy program that facilitates the following movements: grasping, abduction, stretching, strengthening, positioning, splinting, and casting [18]. Rehabilitation is a long-term and demanding process [19].

The literature provides different experiences of using virtual reality (VR) [20-25] and serious games [26-29] that create opportunities for repetitive activities that develop neuroplasticity and learning for motor function rehabilitation.

VR can simulate actions that patients perform in real life. Son and Park [29] have established that “ecological validity” is related to the extent to which the evaluation results are applicable to everyday situations. Using VR for rehabilitation purposes has the advantage of ecological validity, as the technology allows for the reduction of the gap between the clinical environment and the daily environment of the patient.

### Related Work

Ahn [30] presented the combined effects of VR and computer game–based cognitive therapy on the development of visual-motor integration in children with intellectual disabilities. The author used Nintendo Wii consoles and a computerized cognitive rehabilitation program. The results showed a significant difference in visual-motor integration of spatial relation and visual-motor speed, motor-reduced visual perception, and general visual perception. Bortone et al [31] presented the use of VR with a haptic device in a control trial with children with cerebral palsy or developmental dyspraxia to address upper-limb impairments. All children showed significant improvements in the 2 kinesiological measurements (movement error and smoothness) reported.

The study of Şahin et al [32] was designed to investigate the effects of using Microsoft Kinect devices on gross and fine motor functions and activities of daily living (ADL) in children with unilateral spastic cerebral palsy. The results showed that total motor function and total independence in ADL improved after the intervention period of 8 weeks. Faria et al [33] presented a VR-based intervention focused on executive function tasks integrated into ADL involving a virtual simulation of a city.

VR ecological systems have shown significant improvements in rehabilitation by supporting customized and ecologically valid tasks, especially those involving ADL simulations. Our study aimed to analyze the use of an ecological serious game to improve fine motor control in children with EE.

## Methods

### Game Design Process

The game design process involved the following steps. First, the exercises to be performed as the mechanism of interaction with the serious game were selected. Second, the part of the body where the therapy would be applied was identified. Third, whether patients may find it difficult to properly hold or wear physical devices was investigated. Fourth, the game was incrementally developed with continuous communication among developers, neurology specialists, and therapy specialists. Fifth, specialists validated that every increment facilitated the performance or objective of the selected exercise. Sixth, clinical and kinematic evaluations were conducted; the serious game must be clinically validated to establish the effects of the VR therapy and compare them with the effects of standard therapy to determine whether the serious game is appropriate for the motor rehabilitation goal. Finally, user satisfaction was evaluated to discover features to be improved in terms of the control of the game, game information provided, and successful use of the system and even whether there was some discomfort in using the game.

### Game Mechanics

Mechanics (actions, behaviors, and control mechanisms) are controlled by a contactless device leap motion controller (LMC) [34]. LMC detects, recognizes, and captures hand gestures and finger positions in real time with submillimeter precision. The technical features of LMC allow the detection of the required exercises: picking up, grasping, manipulating, and releasing virtual objects using the hand, fingers, and thumb.

### Game Description

**Figure 1:**
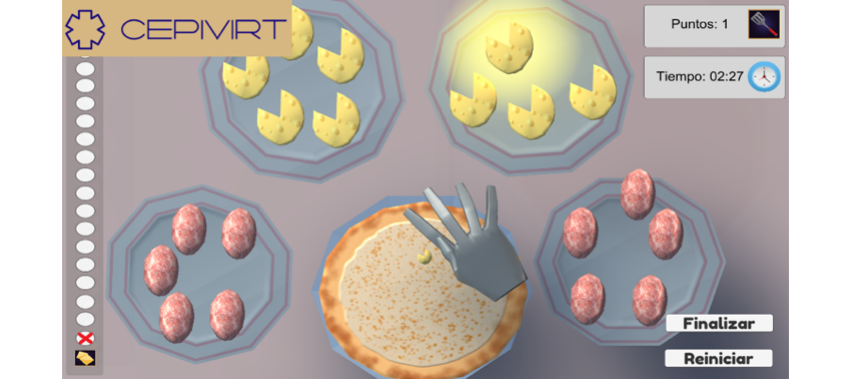
Pizza game interface.

The game is a 2D ecological environment that is composed of 3 modules: participant registration, pizza game, and results. It focuses on performing coordinated actions of handling objects, namely picking up, grasping, manipulating, and releasing the objects using one of their hands. The exercises are based on the International Classification of Functioning, Disability, and Health [35]. To reproduce the hand movements, the game simulates the preparation of a pizza, wherein the participants take 1 highlighted ingredient (virtual object) from a plate and drop it on the pizza dough. The number of ingredients can be 5, 10, 15, or 20. It is possible to select which hand to use (right or left). The game records the time it takes the participants to complete dropping each ingredient on the pizza and the total preparation time (dropping all the ingredients). It also records the number of hits, which are hand movements that accomplish the goal of dropping the ingredients on the pizza, that the participants achieve (Figure 1). In the case of a hit, a bell rings, and in the case of a mistake, a horn sounds.

**Figure 2:**
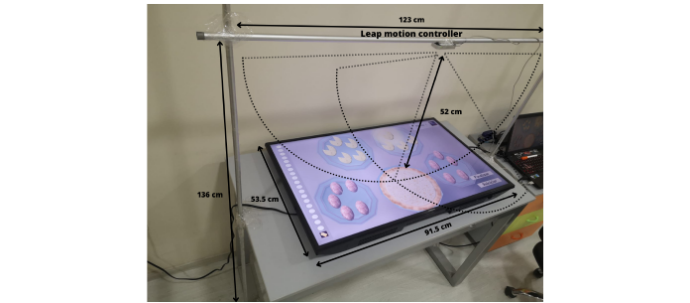
The handling structure and position of the leap motion controller.

Given the pathology of the patients, pediatric neurology has suggested the use of a contactless and nonimmersive technology that uses muted colors (not bright) to avoid the generation of seizures. Therefore, the authors selected LMC, which is a small, contactless optical tracking device that is oriented to gestural hand movement control [34]. To develop a realistic environment, we used a TV screen placed face up on a table to represent a kitchen environment. A simple structure with aluminum bars was designed and built to position the LMC (Figure 2). After several tests, the structure was placed 52 cm above the display screen based on the height of the children and the range of coverage of the LMC.

**Figure 3:**
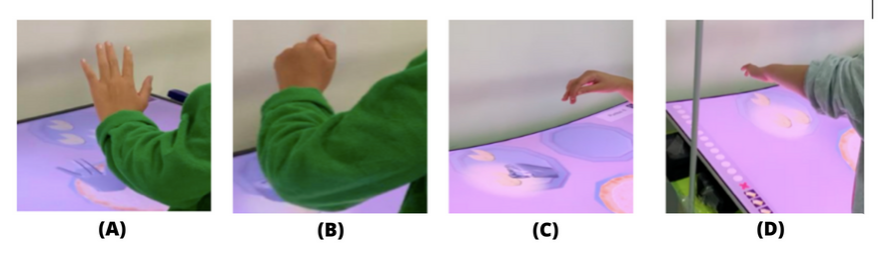
The participants’ hand positions when playing the pizza game.

For the device to recognize the pickup, grasp, manipulation, and release actions, the participants must perform the following sequence: (1) extend their fingers as far as they can, given their condition, which was detected by the system during the initial calibration; (2) close the fingers of the hand as much as the condition allows to grasp the virtual object; (3) move the hand with the ingredient over the pizza; and (4) drop the ingredient on the pizza (Figure 3).

### Recruitment

The inclusion criteria were as follows: (1) sufficient cognitive capacities and aptitudes for the management and understanding of activities performed using technological systems, (2) psychomotor skills that allow the coordination of hand movements to follow the proposed exercises, (3) comprehension of the Spanish language, and (4) informed consent from the parents or legal guardians and agreement to participate in the research. The exclusion criteria were as follows: (1) children with other types of epilepsy and (2) children with EE whose seizure frequency may make it difficult to perform the tasks in this study. All these criteria were confirmed by each child’s pediatric neurologist.

Overall, 4 children who met the inclusion criteria were included in this study. All the children attended all 10 sessions, and there were no absences. The baseline characteristics of the children are presented in Table 1. The mean age of the children was 12.25 (SD 3.4) years, and there were 2 (50%) male and 2 (50%) female children. The children were classified as belonging to level 1 in the Gross Motor Classification System [36].

**Table 1:** Characteristics of the participants in the study.

Participant	Sex	Age (years)	Preferred hand	Clinical condition	GMFCS^a^
1	Male	17	Right	Frequent epilepsy and fine motor problems	Level 1
2	Female	11	Right	Controlled epilepsy and mild cognitive problems without severe motor problems	Level 1
3	Male	12	Right	Controlled epilepsy, mild cognitive problems, and mild motor problems	Level 1
4	Female	9	Right	Frequent epilepsy and fine motor problems	Level 1

^a^GMFCS: Gross Motor Classification System.

### Ethical Considerations

This study was approved by the Ethical Committee of Universidad Cayetano Heredia (reference number: 2008-0234) following the Declaration of Helsinki. Written informed consent was provided by the parents and written informed assent was provided by the participants before the sessions began. The children who visited the research laboratory from January 2023 to February 2023 were assessed for inclusion in the study.

### Instruments

During the intervention period, the children were evaluated by a physical therapist at baseline (before treatment, period A) and immediately after the 10 sessions (after treatment, period B). Outcomes related to fine motor control function were assessed using the Bruininks-Oseretsky Test of Motor Proficiency, 2nd edition (BOT-2) [2], and the Pittsburgh Rehabilitation Participation Scale (PRPS) [37] was used to measure patient participation in rehabilitation sessions. The BOT-2 is a standardized instrument for evaluating motor function in children with disabilities. The BOT-2 assesses proficiency in the motor area in patients aged between 4 and 21 years. To evaluate fine motor control, 2 subscales of the BOT-2 were used, namely FMP and FMI. To evaluate manual coordination, 2 other subscales of the BOT-2 were used, namely manual dexterity (MD) and upper-limb coordination (ULC).

The FMP subscale comprises 7 items. It involves tasks such as coloring shapes, drawing within lines, connecting dots, folding paper, and cutting with scissors and measures how precise the control of finger and hand movements are during these tasks. The FMI subscale comprises 8 items. It requires patients to reproduce drawings of different geometric shapes and measures the integration of visual cues with motor control. The MD test comprises 5 items. It involves tasks that measure fine motor speed and accuracy to capture the dexterity required to complete ADL. Finally, the ULC subscale comprises 7 items. It was designed to measure visual tracking and coordinated arm and hand movements during ball manipulation.

The PRPS is a clinician-rated, 6-point Likert scale that measures patient participation in rehabilitation sessions. The values in this test represent the following: 1 stands for none and indicates that the patient refused the entire session or did not participate in any exercise in the session; 2 stands for poor and indicates that the patient refused or did not participate in at least half of the session; 3 stands for fair and indicates that the patient participated in most or all of the exercises but did not show maximal effort or finish most exercises or that the patient required a lot of encouragement to finish the exercises; 4 stands for good and indicates that the patient participated in all of the exercises with good effort, finished most but not all of the exercises, and passively followed directions; 5 stands for very good and indicates that the patient participated in all of the exercises with maximal effort and finished all of the exercises but passively followed directions; and 6 stands for excellent and indicates that the patient participated in all of the exercises with maximal effort, finished all of the exercises, and actively took interest in the exercises and future therapy sessions [37].

The User Satisfaction Evaluation Questionnaire (USEQ) [38] is a questionnaire designed to evaluate user satisfaction with virtual rehabilitation systems. It comprises the following six questions that can be rated on a Likert scale ranging from 1 (not at all) to 5 (very much): (1) Did you enjoy your experience with the system? (2) Were you successful using the system? (3) Were you able to control the system? (4) Is the information provided by the system clear? (5) Did you feel discomfort during your experience with the system? and (6) Do you think that this system will be helpful for your rehabilitation? It was adapted to evaluate the serious game.

The total score on the USEQ questionnaire ranges from 6 (poor satisfaction) to 30 (excellent satisfaction). The numerical value of the positive questions is used to calculate the total score. The numerical value of the negative question, question 5, is subtracted from the numerical value of question 6, and the resulting value is then added to the total score.

### Intervention

This study included 4 children with EE, and the therapy included ten 30-minute sessions involving the game twice a week over a period of 5 weeks in the research laboratory under the supervision of a physical therapist. Before the first session, the following steps were carried out: (1) the system was configured based on the initial condition of the participants (indicated by the physical therapist); (2) the initial state of the participants was evaluated using the BOT-2 instrument; and (3) the participants’ personal information was stored, and the clinical specialist explained the correct movements of the hand to the participants to enable them to interact with the game. During each session, the patients used the system first with their right hand and then with their left hand, moving only 5 virtual objects each time. Then, the participants took a 5-minute break. Finally, they moved 10 virtual objects with their left or right hand. The system recorded the duration of each interaction as well as the number of hits or faults.

The physical therapist evaluated the degree of patient participation in each session using the PRPS. In addition, at the end of the first session, the clinical specialist administered the USEQ. After the 10th session, the physical therapist evaluated the final state of the patient using the BOT-2 clinical test.

## Results

### Kinematic Outcomes

**Figure 4:**
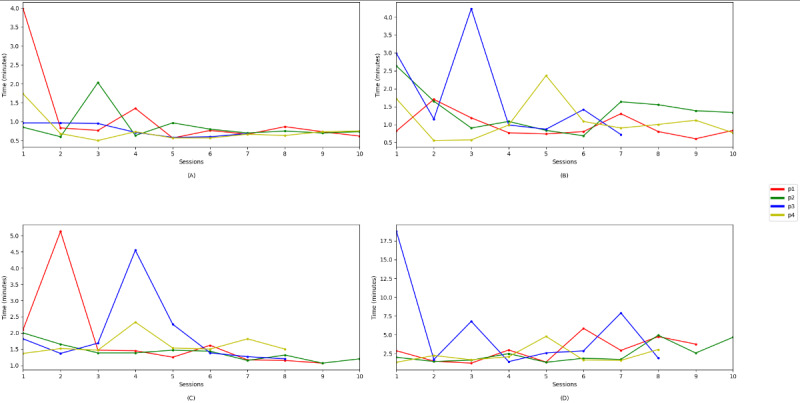
Kinematic outcome: duration spent on each interaction by each participant (p). Tracking time: (A) right hand, 5 elements; (B) left hand, 5 elements; (C) right hand, 10 elements; and (D) left hand, 10 elements.

Figure 4 presents the kinematic outcomes of our study, which focused on the left- and right-hand movements executed by the participants when interacting with the game and the duration of each interaction.

### Statistical Analysis

Statistical analysis was carried out in the SPSS software (IBM Corp). The statistical analysis included a Student *t* test (1-tailed) for samples related to the Shapiro-Wilk normality test. The significance results for the Student *t* test are as follows: *P*=.009 for FMP, *P*=.002 for FMI, *P*=.56 for MD, and *P*=.99 for ULC. As *P*<.05 for FMP and FMI, there was a significant difference in the means before and after treatment. This means that the game had significant effects. These results are shown in Table 2.

**Table 2:** Bruininks-Oseretsky Test of Motor Proficiency, 2nd edition (BOT-2) outcomes and statistical analysis.

Subscale and time point			Participant 1		Participant 2		Participant 3		Participant 4		*P* value	
**FMP^a^**												.009
	Before treatment	23		17		28		13				
	After treatment	30		26		32		22				
**FMI^b^**												.002
	Before treatment	12		16		27		12				
	After treatment	16		27		31		21				
**MD^c^**												.56
	Before treatment	16		7		12		8				
	After treatment	10		8		23		11				
**ULC^d^**												<.99
	Before treatment	3		7		10		8				
	After treatment	5		7		7		8				

^a^FMP: fine motor precision.

^b^FMI: fine motor integration.

^c^MD: manual dexterity.

^d^ULC: upper-limb coordination.

### PRPS Outcomes

**Figure 5:**
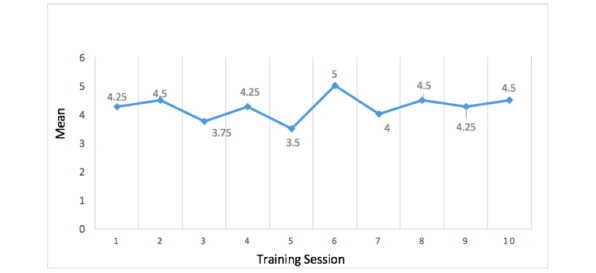
Mean Pittsburgh Rehabilitation Participation Scale (PRPS) scores of all the participants after each training session.

The physical therapist evaluated the degree of patient participation in each session using the PRPS, the outcomes of which are shown in Figure 5.

### USEQ Outcomes

The outcomes of the USEQ questionnaire, which was administered at the end of the first session, are shown in Table 3.

**Table 3:** User Satisfaction Experience Questionnaire outcomes.

Item	Values, mean (SD)
Question 1: Did you enjoy your experience with the game?	4.75 (0.5)
Question 2: Were you successful using the game	4.50 (1.0)
Question 3: Were you able to control the game?	4.25 (0.96)
Question 4: Is the information provided by the game clear?	4.50 (0.58)
Question 5: Did you feel discomfort during your experience with the game?	1.25 (0.5)
Question 6: Do you think that this game will be helpful for your rehabilitation?	4.75 (0.5)
Total score	27.50 (4.035)

## Discussion

### Principal Findings

Virtual rehabilitation environments typically have goals to attain that become progressively more difficulty [19]. Working to achieve a goal may enhance attention and concentration in therapy, potentially increasing the efficacy of rehabilitation interventions [39,40]. Working toward a goal of achieving a high score in a virtual environment may enhance children’s enjoyment of therapy. We believe that therapy using serious games can mimic everyday situations and recreate the real world with varying degrees of accuracy. The significance outcomes for the Student *t* test were as follows: *P*=.009 for FMP, *P*=.002 for FMI, *P*=.56 for MD, and *P*=.99 for ULC. The participation rate as measured using the PRPS was between good and very good, which means that, based on the therapist’s evaluation, interest, independence, and motivation were achieved by each participant. The mean USEQ score was close to 30, which is the maximum score attainable on the questionnaire.

Regarding the kinematic outcomes, for the interactions using the right hand with 5 virtual objects (Figure 4A), there was a clear reduction in duration; the longest duration (4 min) spent was in session 1, and the shortest duration (<1 min) spent was in session 10 for all 4 participants. Similarly, for the interaction using the right hand with 10 elements (Figure 4C), there was a reduction in duration throughout the sessions, except for 2 long durations spent by participants 1 and 3 in sessions 2 and 3, respectively. In both cases, the values were within an almost constant range.

For the interactions using the left hand with both 5 and 10 virtual objects (Figures 4B and 4D), there were greater reductions in duration. This is explained by the fact that, for the 4 participants, the dominant hand was the right hand. A considerable reduction in duration was observed from sessions 1 to 10.

These results demonstrate initial progress in MD, which measures fine motor speed and accuracy to capture the dexterity required to complete ADL. This is because the game allows the participants to concentrate on the action to be carried out through the virtual object that is highlighted, which must be selected to drop it on the pizza dough.

Regarding the statistical analysis, the results in Table 2 show that the 4 participants showed improvements in their assessed proficiency in fine motor control after receiving a 10-session intervention. Dependent *t* tests confirmed the observations in the FMP and FMI subtests.

The results for manual coordination showed that 1 (25%) of the 4 participants showed significant improvements in the MD subtest (*P*=.009 for FMP, *P*=.56 for MD, and *P*=.99 for ULC). With respect to ULC, 2 (50%) participants showed no improvement during the intervention period, and only 1 (50%) participant showed a slight improvement. Given that the system’s focus is centered on using only 1 hand at a time oriented to the repetition of tasks (according to the game setting, there can be 5, 10, 15, or 20 ingredients; the participant has to execute the same exercise to get the objective done), coordination exercises using both hands such as passing items from one hand to another, separating items into stacks, or inserting items onto a string were not reinforced for ADL.

The lack of this type of training was reflected in the results of the MD subtest. The goal-oriented nature of many tasks in serious games may enhance cognitive engagement with the tasks and thus their salience [41].

The participation rate as measured using the PRPS was between good and very good, which means that, based on the therapist’s evaluation, interest, independence, and motivation were achieved by each participant. We believe that the game helped to increase motivation, as it was fun for the children and was of interest to them because it was ADL oriented and the participants got points each time they put the virtual object on the pizza. Our results are in accordance with those of the study by Holden [40], which affirmed that VR is a powerful tool for providing participants with an opportunity to engage in repetitive practice, feedback about their performance, and motivation to reinforce practice.

The mean USEQ score was close to 30, which was the maximum score attainable on the questionnaire. These results show that the patients enjoyed the experience of playing the game and found it easy to play. We believe that these results are because the system is ecological, consistent with the study by Nir-Hadad et al [42], which stated that the use of VR in rehabilitation enables the simulation of various real-life situations. LMC creates an interface for motor rehabilitation that is natural and does not require physical contact. This feature was ad hoc for participants with EE. LMC is a good alternative for serious games for the improvement of coordination, speed of movements, and fine upper-limb dexterity [43]. The outcome of the USEQ reinforces this statement.

### Comparison With Prior Work

Bui et al [44] argued that training based on VR can provide an almost natural and ecologically valid environment. Arlati et al [45] suggested that having a more ecological environment would favor the generation of more natural behaviors and, consequently, movements with kinematic similarity.

Another important study whose results support ours is the study by Mota et al [46], which states that a serious game that is very similar to real life and produces a direct interaction between the player and the virtual objects has ecological validity and ensures that the player is comfortable when performing the tasks [46].

The participants showed improvements in fine motor control (as measured via FMP and FMI subtests). The proposed serious game promotes the repetition of interactions with 5 and 10 virtual objects in each training session. Our findings are in agreement with those of Montoro-Cárdenas et al [47] and Palsbo et al [48]. They determined that the use of VR technology induces neuroplasticity and causes changes in the cerebral cortex, as demonstrated by neuroimaging studies that were conducted in children with cerebral palsy, and that training with repetitive movements increases the connection of new neural pathways.

### Strengths and Limitations

Feedback is classified into feedback on performance (information about how a person performed a movement) and feedback on results (information about whether the movement produced the desired goal) [49]. The game is oriented toward the second type of feedback because it focuses on dropping each ingredient on the pizza. Working to achieve this goal may enhance attention and concentration in therapy, potentially increasing the efficacy of rehabilitation interventions [40].

The exercises that the game focuses on are the coordinated actions of picking up, grasping, manipulating, and releasing objects using one of the hands. In addition, the game provided opportunities for performing repetitive tasks, simulating real-world movements related to ADL. The intervention used in this study placed more emphasis on repeated practicing of reaching movements in horizontal directions, which was reflected by the improvements in FMP and FMI subtests.

The evaluation was carried out considering only the hits (the hand movements that produced the desired goal) throughout the sessions. It has been observed that across the sessions, the number of hits increased (or the number of faults reduced). We also believe that motivation played an important role, as the participants received visual and auditory feedback each time they achieved a hit. All these components are important for improving fine motor control skills.

This study has a few limitations. First, as the sample size was small, one must be cautious when interpreting the statistical results. The main concern of having a small sample size is that we cannot generalize the results. Likewise, having 4 patients with dominant right hands did not allow us to evaluate the effectiveness of fine motor control in the left hand. In addition, although the usability results were satisfactory, with a larger sample, it is possible to obtain different results when testing the feasibility of the game for improving fine motor control. The sample size in our study was small because the pathology treated is considered a rare disease, with a very low incidence and prevalence. Future studies should be conducted with a sufficiently representative sample size for statistical analyses. Second, we used only a specific device, LMC. In a future work, we plan to try other contactless devices. Third, the game does not have exercises aimed at visual coordination tracking and coordinated arm and hand movements.

### Future Directions

We seek to evaluate the impact of the serious game on the improvement of gross and fine motor disorders in a future work. The aim is to create more nonimmersive ecological serious games for children with other pathologies who seek improvement at both gross and fine motor levels. In addition, we believe that this game could be complementary to fine motor rehabilitation considering the following benefits: (1) it can be undertaken at home, which might make the activity more comfortable; (2) the game will provide access to perform the rehabilitation exercises; (3) it allows the tracking of the player’s progress, which could be evaluated by the therapist on the web; and (4) the provided feedback could reduce the need for continuous supervision by the therapist. There is a strong opportunity to have a home-based fine motor rehabilitation game. To implement it at home, the potential users only need a computer to install the game, the LMC (which is a low-cost device), and a TV screen. The special structure we have developed to place the LMC above the TV screen could also be used to place the LMC below the TV screen.

### Conclusions

In this study, we explored the feasibility of a new serious game that serves as a complement to traditional rehabilitation processes to improve fine motor control in children with EE. The results show significant improvements in fine motor control, alleviating the symptoms associated with EE. These types of studies encourage us to continue creating new ecological serious games that are focused on the existing rehabilitation processes.

We can conclude that the proposed serious game could help improve fine motor precision and FMI in pathologies of this type or similar types that present alterations in fine motor control.

## Abbreviations

ADL: activities of daily living
BOT-2: Bruininks-Oseretsky Test of Motor Proficiency, 2nd edition
EE: epileptic encephalopathy
FMI: fine motor integration
FMP: fine motor precision
LMC: leap motion controller
MD: manual dexterity
PRPS: Pittsburgh Rehabilitation Participation Scale
ULC: upper-limb coordination
USEQ: User Satisfaction Evaluation Questionnaire
VR: virtual reality

## Data Availability

The data sets generated during this study are available in the Data01_CEPIVIRT repository [50].
